# Effects of aging and dual tasking on step adjustments to perturbations in visually cued walking

**DOI:** 10.1007/s00221-015-4407-5

**Published:** 2015-08-23

**Authors:** Masood Mazaheri, Wouter Hoogkamer, Zrinka Potocanac, Sabine Verschueren, Melvyn Roerdink, Peter J. Beek, C. E. Peper, Jacques Duysens

**Affiliations:** Department of Human Movement Sciences, MOVE Research Institute Amsterdam, VU University Amsterdam, Amsterdam, The Netherlands; Department of Kinesiology, KU Leuven, Leuven, Belgium; Department of Rehabilitation Sciences, KU Leuven, Leuven, Belgium

**Keywords:** Step adjustment, Aging, Dual tasking, Attention, Visually guided walking

## Abstract

Making step adjustments is an essential component of walking. However, the ability to make step adjustments may be compromised when the walker’s attentional capacity is limited. This study compared the effects of aging and dual tasking on step adjustments in response to stepping-target perturbations during visually cued treadmill walking. Fifteen older adults (69.4 ± 5.0 years; mean ± SD) and fifteen young adults (25.4 ± 3.0 years) walked at a speed of 3 km/h on a treadmill. Both groups performed visually cued step adjustments in response to unpredictable shifts of projected stepping targets in forward (FW), backward (BW) or sideward (SW) directions, at different levels of task difficulty [which increased as the available response distance (ARD) decreased], and with and without dual tasking (auditory Stroop task). In both groups, step adjustments were smaller than required. For FW and BW shifts, older adults undershot more under dual-task conditions. For these shifts, ARD affected the age groups differentially. For SW shifts, larger errors were found for older adults, dual tasking and the most difficult ARD. Stroop task performance did not differ between groups in all conditions. Older adults have more difficulty than young adults to make corrective step adjustments while walking, especially under dual-tasking conditions. Furthermore, they seemed to prioritize the cognitive task over the step adjustment task, a strategy that may pose aging populations at a greater fall risk. For comparable task difficulty, the older adults performed considerably worse than the young adults, indicating a decreased ability to adjust steps under time pressure.

## Introduction

The study of mechanisms behind the occurrence of falls in elderly has recently received a lot of attention. In an observational study, based on video footage of real-life falls, it has been shown that the most frequent cause of falling (accounting for 41 % of falls) is an incorrect shift in body weight (including, for instance, misplaced steps during walking; Robinovitch et al. 2013). This finding highlights the importance of assessing behaviors like step adjustments during walking, which are highly dependent on weight shift strategies.

Step adjustments to sudden shifts in stepping targets have been studied extensively in relation to step initiation from standstill situations (Melzer and Oddsson 2004; Reynolds and Day 2005; Tseng et al. 2009; Melzer et al. 2010; Kim and Brunt 2013). This complex behavior requires two integrated motor skills: control of foot trajectory and control of balance (Reynolds and Day 2005; Tseng et al. 2009). While step initiation requires preplanning of foot placement and the associated postural adjustment, the shift of a stepping target after the step initiation stresses the need to modify these preplanned actions. Healthy young adults have shown fast and accurate step adjustments to unpredictable stepping-target shifts during step initiation without compromising balance (Reynolds and Day 2005; Tseng et al. 2009). However, this ability can be affected with increased age (Tseng et al. 2009; Young and Hollands 2012; Kim and Brunt 2013). That is, older adults have shown delayed onset of foot trajectory modification and prolonged execution time of the stepping limb in response to sudden stepping-target shifts during step initiation (Tseng et al. 2009). Tseng and colleagues (2009) related these deficits to degraded postural reactions of the contralateral leg, as evidenced by delays and decreases in the ground reaction force of the stepping response (see also Kim and Brunt 2013). Most of these deficits become more prominent with decreased available response time (Tseng et al. 2009; Kim and Brunt 2013). This means that in contrast to young people, older adults are less able to speed up reactive step adjustments under increased time pressure conditions (Tseng et al. 2009). These deficits might prevent timely step adjustments, and their association with falls has been investigated previously. For example, Melzer and colleagues (2010) have shown that older recurrent fallers exhibit slower step adjustments compared to older non-fallers.

The aforementioned findings, however, are related to step initiation when stepping targets are suddenly perturbed in medial and/or lateral direction. As most falls occur during walking (Robinovitch et al. 2013), these step initiation experiments hardly reflect the step adjustment behaviors required during walking. Recent studies on walking adaptability or gait adaptability, i.e., the ability to adjust walking to meet task goals and environmental demands (Houdijk et al. 2012; Balasubramanian et al. 2014), presented visual context onto a walking surface in the form of obstacles (Potocanac et al. 2013) or stepping targets (Mazaheri et al. 2014). In this way, step adjustments can be elicited by introducing shifts in stepping-target locations in different directions under various time pressure conditions (Bank et al. 2011; Peper et al. 2012; Young and Hollands 2012), which allows examining the ability to make step adjustments during walking. Another factor that limits the generalizability of step adjustment experiments (in both standstill and walking situations) to real-life situations is their focus on single-task conditions, while in reality step adjustments often occur while attention is shared with a secondary task, such as talking. The effect of a concurrent attention-demanding task on voluntary step initiation (with no target shift) has been examined in previous studies (Melzer and Oddsson 2004; St George et al. 2007). It was concluded that older people, in particular those at risk of falling, have an impaired ability to make accurate voluntary steps, especially when performing a dual task concurrently. To our knowledge, no studies to date have determined the effects of a concurrent attention-demanding task on step adjustments in response to sudden stepping-target shifts during walking.

Therefore, this study was designed to investigate the effect of age and dual tasking on step adjustment in response to unpredictable stepping-target shifts in different directions (i.e., forward, backward or sideward) under various time pressure conditions during visually cued treadmill walking. The feasibility of such tests was demonstrated in previous studies (Bank et al. 2011; Peper et al. 2012; Hoogkamer et al. 2015). In the current study, the step adjustment task was performed with and without an auditory Stroop task (which served as a concurrent attention-demanding task). The primary outcome measure was the step adjustment error, whereas the accuracy and reaction time on the Stroop task served as important secondary outcome measures for exploring task prioritization effects. It was hypothesized that all outcome measures would deteriorate with increased task difficulty (i.e., with increased time pressure demands). Moreover, it was expected that dual tasking has a detrimental effect on the step adjustments and that this influence is more prominent in older adults than in young adults.

## Materials and methods

### Participants

Fifteen healthy older adults (female/male: 10/5; mean ± SD age: 69.4 ± 5.0 years; weight: 67.6 ± 6.9 kg; height: 165.2 ± 7.4 cm) and fifteen young adults (female/male: 10/5; age: 25.4 ± 3.0 years; weight: 66.3 ± 9.0 kg; height: 173.0 ± 8.2 cm) participated. Participants had no self-reported cardiovascular or cardiopulmonary problems, orthopedic conditions, uncorrected visual or auditory impairments, neurological disorders, or other conditions limiting mobility; they did not use a walking aid and were able to speak Dutch. All older adults had a Mini Mental State Exam score above 19 (actual range 27–30). Thirteen older adults had no history of falls, one reported one fall, and one reported two falls over the last year. The local ethics committee approved the experiment. All volunteers provided written informed consent before participating in the study.

### Setup

Participants walked on a force platform instrumented treadmill (custom-built, ForceLink, Culemborg, The Netherlands) equipped with a projector and C-Mill software (Cuefors, ForceLink, Culemborg, The Netherlands), allowing the projection of stepping targets onto the belt’s surface based on online detected gait events (using center of pressure (COP) data, sampled at 1000 Hz; Roerdink et al. 2008). In addition, reflective markers were attached to both shoes with two markers mounted on each shoe along the AP axis of the foot at heel side (at the approximate position of the calcaneal tuberosity) and toe side (at the approximate position of the second toe) to record stepping errors relative to the projected targets, using a 10-camera Vicon motion capture system (Oxford Metrics Group, Oxford, UK) at 100 Hz. During the dual-task trials, participants wore a headphone and a head-mounted microphone (wireless recording at 3000 Hz).

### Procedure

#### Step adjustment task

**Fig. 1:**
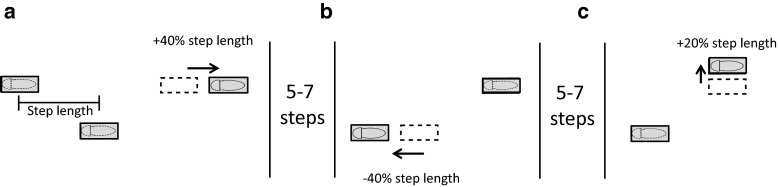
Schematic of the stepping-target shifts in the three directions. The distance between the stepping targets was determined based on participant’s preferred step length. At a random time instant, a single stepping target (indicated by *dashed squares*) was shifted in either forward (size: 40 % of step length), backward (size: 40 % of step length) or sideward (size: 20 % of step length) direction which required, respectively, a longer-step (**a**), a shorter-step (**b**) or a side-step (**c**) response. The shift targeted either the right or left leg. At least five and at most seven non-shifted stepping targets were presented between the shifted targets

Participants walked at 3 km/h on the treadmill.^1^ The speed at which the stepping targets approached the participant was equal to the belt speed, which was constant across the experiment. The size of the stepping targets was adjusted to the participant’s shoe length and width. The anterior–posterior (AP) distance between the stepping targets was attuned to the participant’s preferred step length (determined by the C-Mill software from COP data (Roerdink et al. 2008) based on 20 s of uncued walking). The mediolateral (ML) distance between the stepping targets was 20 cm. The participants were instructed to place their feet as accurately as possible on the stepping targets. Every now and then, a stepping target would shift and participants needed to adjust their step to the new target location. These shifts targeted either the right or left leg, with 5–7 non-shifted targets in between the target shifts. Stepping stones were unpredictably shifted in different directions, i.e., forward (FW), backward (BW) or sideward (SW), in the same way as in Hoogkamer et al. (2015). Longer-step responses were required for FW shifts, shorter-step responses for BW shifts and side-step responses for SW shifts. The size of the stepping-target displacement was scaled to the individual’s preferred step length: 40 % for FW and BW shifts and 20 % for SW (Fig. 1). The smaller SW shifts were motivated by the observed preference for adjustments in the plane of progression as opposed to the frontal plane (Patla et al. 1999). Because lateral step adjustments are more successful than medial ones (Moraes et al. 2007), the SW shifts were presented in the lateral direction only. The stepping-target shifts occurred when the approaching stepping target came within a threshold distance from the participant’s COP. This ‘available response distance’ (ARD) was set to 130, 110 or 90 % of the preferred step length, and the lower it was, the more difficult the task was. These individualized ARDs were selected based on success rates during previous experiments using the same experimental setup (Potocanac et al. 2013; Hoogkamer et al. 2015).

#### Auditory Stroop task

Through the headphone, a series of ‘high’ and ‘low’ words spoken in Dutch was presented, in either high-pitched or low-pitched voice (McClain 1983). Both congruent (where the word and the vocalized pitch matched) and incongruent (where the word and the vocalized pitch differed) stimuli were presented randomly with an inter-stimulus interval of 1.6 s. The participants were asked to name the pitch of the voice as accurately and as quickly as possible (recorded by means of the microphone).

#### Protocol

Participants were first familiarized with treadmill walking for 5 min. After determination of preferred step length, participants were gradually introduced to the experimental tasks by means of walking on a series of 20 stepping targets presented to cue the steps of both legs without target shifts and then on a second sequence of 120 stepping targets, of which 18 (9 per leg) shifted in different directions.

The experiment consisted of two blocks (single task and dual task), each comprising three step adjustment ARD conditions (130, 110 or 90 % of preferred step length), which were presented randomly within both blocks. Each ARD condition involved 60 shifted stepping targets of which 20 (10 per leg) shifted in FW, 20 (10 per leg) shifted in BW and 20 (10 per leg) shifted in SW direction. The shift directions were randomly distributed within each trial. The single-task block was presented first, and after a 10-min break, participants were gradually prepared to perform the second block in which the walking task was performed concurrently with the auditory Stroop task. In preparation for the dual-task block, participants were first acquainted with the auditory Stroop task while seated. Further practice of the Stroop task was performed during walking on a series of 20 stepping targets followed by walking on a second sequence of 120 stepping targets, of which 18 (9 per leg) shifted in different directions. Additionally, the dual-task block included a condition of cued walking without stepping-target shifts, which served as the baseline condition for the auditory Stroop task. This condition was randomized together with the other conditions. In the dual-task conditions, participants were asked to give equal emphasis to both tasks. The total experiment took about 2 h.

### Data analysis

Stepping error was defined as the median of the AP distance (for FW and BW shifts) or the ML distance (for SW shifts) between the center of a shifted stepping target and the center of the foot at midstance (at 50 % of the time between heel strike and toe-off). The location of the center of each stepping target at midstance was available from the C-Mill software. The center of the foot at midstance was derived from the Vicon data. The foot was defined as the line connecting the reflective markers at the heel and the toe. The center of the foot was at 50 % of this line. Stepping error was corrected for stepping bias by subtracting the distance between the foot and the stepping target at midstance as obtained for the last step before the shifted stepping target. Finally, the bias-corrected stepping error was normalized to each participant’s preferred step length. Negative stepping error values indicated undershooting the shifted target.

The auditory Stroop task data were analyzed using a computerized analysis program (Potocanac et al. 2015), which was fine-tuned to a given participant’s data. The software extracted the spoken words from the continuous audio recording of the trial by means of a threshold and recognized the content of the spoken words based on the mel-frequency cepstral coefficients matched to Gaussian mixture models of signals for ‘high’, ‘low’ and noise made previously. Accuracy of the extracted word recognition, evaluated by tenfold cross-validation of a learning set, was 96 %. Accuracy of the pitch analysis was 100 %. Response latency was defined as the time between stimulus and response onsets. Mean response latency and mean percentage of accurate responses (%Accuracy) were determined for congruent and incongruent stimuli separately. All analyses were performed using MATLAB 2011 (Mathworks, Natick, MA, USA). The data of one older participant were excluded from further analysis in view of a large number of incorrect responses due to insufficient understanding of the Stroop task.

### Statistical analysis

Independent *t* test was used to compare the preferred step length between the two groups. AP stepping error was analyzed using a 2 (group: young vs. older adults) × 2 (cognitive loading: single vs. dual task) × 2 (direction: FW vs. BW) × 3 (task difficulty: ARD 130 vs. 110 vs. 90 %) mixed-model ANOVA with group as between-subject factor and cognitive loading, direction and task difficulty as within-subject factors. The stepping error for SW shifts was obtained in the ML direction and subjected to a separate 2 (group: young vs. older adults) × 2 (cognitive loading: single vs. dual task) × 3 (task difficulty: ARD 130 vs. 110 vs. 90 %) mixed-model ANOVA.

Response latency on the Stroop task was analyzed using a 2 (group: young vs. older adults) × 2 (congruency: congruent vs. incongruent stimuli) × 4 (task difficulty: no-shift vs. ARD 130 vs. 110 vs. 90 %) mixed-model ANOVA with group as between-subject factor and congruency and task difficulty as within-subject factors. Following logarithmic transformation to meet the assumption of normally distributed data, the same ANOVA was applied to the percentage of accurate responses. Significance was assumed for *p* < 0.05. Post hoc pair-wise comparisons were Bonferroni corrected.

## Results

### Stepping error for forward and backward shifted stepping targets

**Fig. 2:**
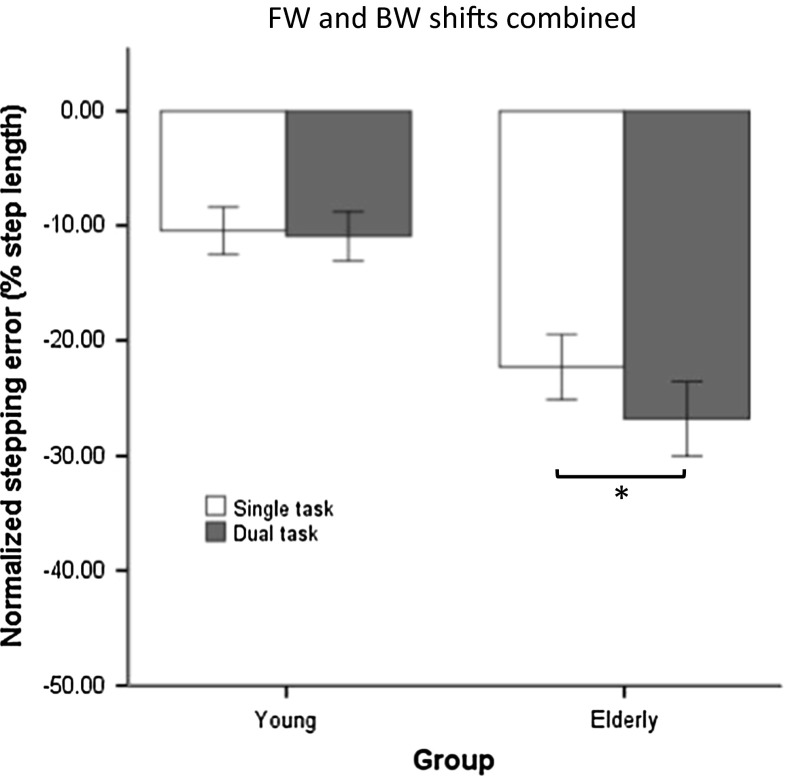
Stepping error following FW and BW shifts (averaged over all difficulty levels) as obtained for the single-task and dual-task conditions in young and older adults. Stepping error values closer to zero indicate better stepping performance. Negative values indicate undershooting the shifted target. *Error bars* indicate standard error of the mean. *Asterisks* indicate significant differences

**Fig. 3:**
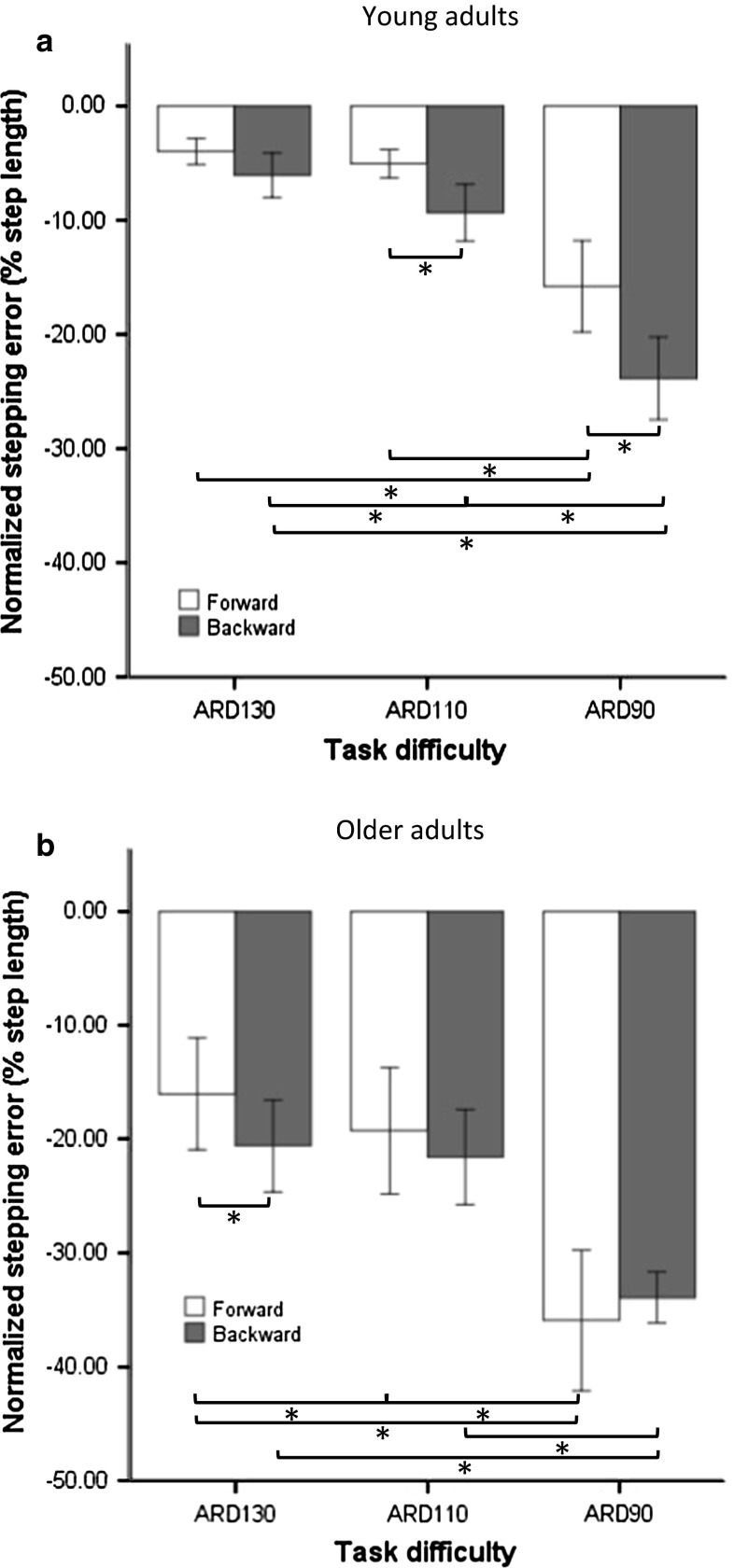
Stepping error following FW and BW shifts across the three levels of ARD as obtained for young (**a**) and older (**b**) adults

The preferred step length was slightly lower for older adults (51.1 ± 6.2 cm; mean ± SD) than for young adults (54.1 ± 3.2 cm), but this difference was not significant (*t*_27_ = 1.63, *p* = 0.12). Stepping errors were negative overall, implying that the shifted targets were undershot. For FW and BW shifts, the significant main effects of group (*F*_1,27_ = 25.01, *p* < 0.001), cognitive loading (*F*_1,27_ = 11.38, *p* < 0.01), direction (*F*_1,27_ = 9.96, *p* < 0.01) and task difficulty (*F*_2,54_ = 118.78, *p* < 0.001) implied a larger error for older adults, for dual tasking, for BW shifts and for more difficult ARD, respectively. For older adults, the concurrent performance of the auditory Stroop task resulted in a larger stepping error compared to single-task conditions, whereas for young adults dual tasking had no effect on stepping error [Fig. 2, illustrating the significant group × cognitive loading interaction (*F*_1,27_ = 7.30, *p* < 0.05)]. The interaction of group × direction × task difficulty was also significant (*F*_2,54_ = 9.02, *p* < 0.001). Post hoc analysis showed larger stepping errors for older adults compared to young adults in both directions and at all difficulty levels. Young adults made smaller step adjustments (i.e., larger errors) to BW shifts compared to FW shifts in the most difficult tasks (ARD 90 % and 110 %; Fig. 3, panel a), whereas for older adults this difference was only observed for the largest (least difficult) ARD (130 %; Fig. 3, panel b). Furthermore, young participants’ performance on BW shifts was more adversely affected by increasing task difficulty compared to FW shifts (Fig. 3, panel a). For them, stepping errors to BW shifts increased significantly from ARD 130 % to ARD 110 % to ARD 90 %, whereas for FW shifts only ARD 90 % differed significantly from the other difficulty levels. In contrast, in older adults the increased stepping error induced by a decrease in ARD was more prominent for FW shifts than for BW shifts (Fig. 3, panel b). For older adults, the changes in stepping error from ARD 130 % to ARD 110 % to ARD 90 % were all significant following FW shifts, while for BW shifts only ARD 90 % differed significantly from the other two difficulty levels.

### Stepping error for sideward shifted stepping targets

**Fig. 4:**
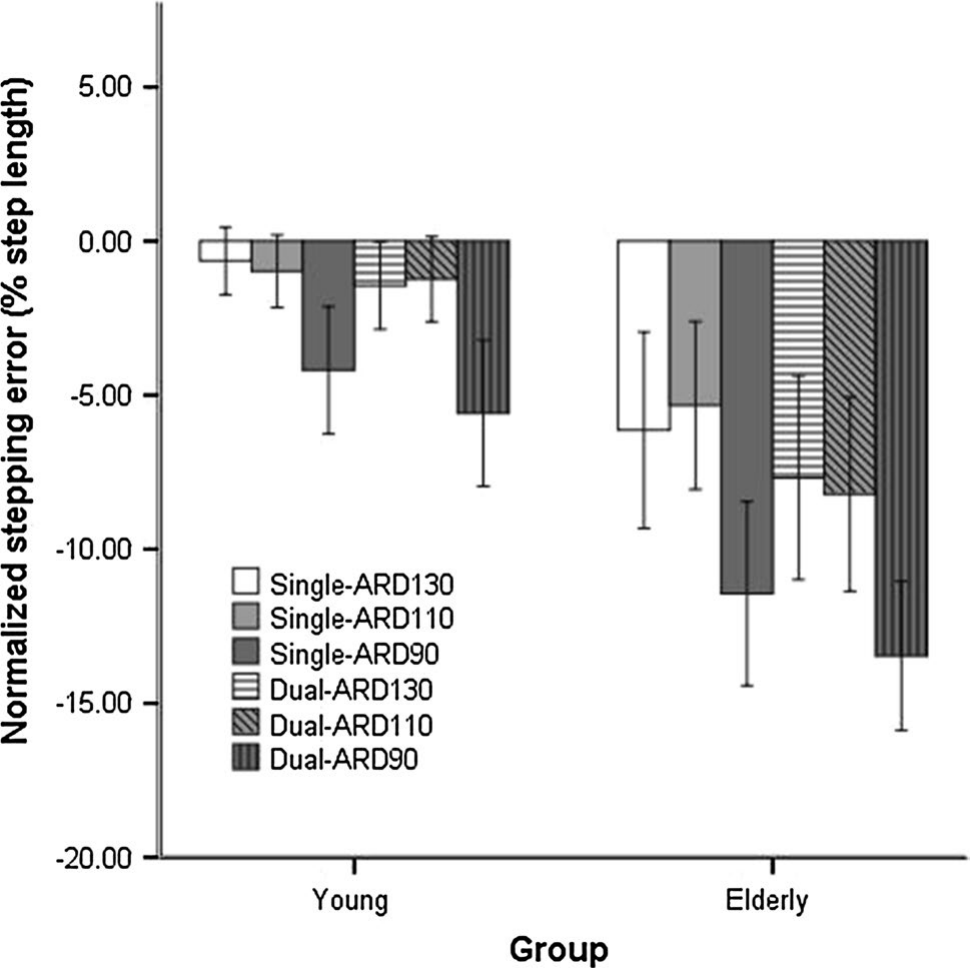
Stepping error following SW shifts across the three levels of ARD in young and older adults. See text for a specification of the statistical results

The ANOVA on stepping error in response to SW shifts showed significant main effects of group (*F*_1,27_ = 23.63, *p* < 0.001), cognitive loading (*F*_1,27_ = 14.83, *p* < 0.01) and task difficulty (*F*_2,54_ = 55.89, *p* < 0.001). Stepping errors were negative overall, implying that the SW shifted targets were undershot. Stepping errors were larger for older adults (−8.7 ± 5.8 %) compared to young adults (−2.3 ± 3.5 %) and for dual-task conditions (−6.2 ± 5.9 %) compared to single-task conditions (−4.7 ± 5.4 %; Fig. 4). Larger errors were found for the most difficult level of ARD (90 %: −8.5 ± 5.8 %) compared to the other two levels (110 %: −3.8 ± 4.9 %; 130 %: −3.9 ± 5.1 %).

### Auditory Stroop task performance

The significant main effects of task difficulty (*F*_3,81_ = 8.64, *p* < 0.001) and congruency (*F*_1,27_ = 35.25, *p* < 0.001) indicated longer response latencies for the two most difficult levels of ARD (90 %: 803 ± 115 ms; 110 %: 787 ± 108 ms) compared to the baseline no-shift condition (742 ± 105 ms) and for incongruent (807 ± 113 ms) compared to congruent (747 ± 101 ms) stimuli. Main or interaction effects involving the factor group were not significant.

For the percentage of correct responses (%Accuracy), a significant main effect of congruency (*F*_1,27_ = 6.35, *p* = 0.02) was found, indicating deteriorated performance with incongruent (89.6 ± 13.8 %) compared to congruent (95.9 ± 4.4 %) stimuli. Again, main or interaction effects involving the factor group were not significant.

## Discussion

In the present study, step adjustments were elicited by means of sudden positional shifts of stepping targets during visually cued treadmill walking. Both age groups could perform this task successfully, although the step adjustments were smaller than the actual target shifts. For shifts in FW and BW direction, this effect was further exacerbated in older adults if they concurrently performed an auditory Stroop task. Whereas for the older participants step adjustment performance significantly decreased as a result of dual tasking, this was not the case for the young participants (Fig. 2). Since the performance of the Stroop task did not differ between the two age groups, this result suggested that the older adults prioritized the auditory Stroop task over the step adjustment task, despite the explicit instruction to give equal emphasis to both tasks. This observation is at odds with the ‘posture-first’ principle (Bloem et al. 2001), according to which individuals prioritize walking over other concurrent tasks in challenging situations. Such optimization of walking at the expense of a secondary task is frequently observed in young adults (Bloem et al. 2006) and is an effective strategy to avoid falls. In contrast, our older adults appeared to be less inclined to use this strategy. They sacrificed their performance on the walking task in order to improve their cognitive task performance. The decline of this ‘posture-first’ strategy may pose aging populations at a greater risk of falling in complex multitask environments (Bloem et al. 2006; Schaefer et al. 2015). Although this result seems to contradict previous work on obstacle avoidance (Potocanac et al. 2015), it is useful to note that the cost of failing to avoid an obstacle (possibly resulting in tripping and falling) is much higher than the cost of missing a shifted stepping target during visually cued treadmill walking. This difference suggests task dependency in the adherence to the posture-first principle.

In this context, it is useful to note that the apparent absence of a posture-first strategy may have been the result of a parsimonious attempt to simply ignore the shifted targets, so as to be accurate on all subsequent unshifted targets (i.e., the vast majority of stepping targets). In this way, essentially no step adjustment is required, instead of two (first to step on the shifted target and then back to step on the subsequent unshifted targets). Perhaps, older adults adopted such a strategy, which would be consistent with the observed large undercorrection, especially under dual-task conditions (Fig. 2). If so, then this strategy may still classify as posture first.

Effective use of step-lengthening and step-shortening strategies to overcome challenges in the environment has been shown to be a critical determinant of safe walking (Chen et al. 1994). The current stepping error results for FW and BW target shifts showed differences between the two age groups with respect to the two types of step adjustment strategies. In young participants, the stepping error to FW shifts was smaller than their stepping error to BW shifts at all ARD levels, implying that longer-step strategies were more effective than shorter-step strategies. This result corresponds to the findings of Hoogkamer et al. (2015), who also observed that, in response to target shifts, longer-step adjustments were performed more successfully than shorter-step and side-step adjustments. In the obstacle avoidance literature, the advantage of the longer-step strategy over the shorter-step strategy has been discussed from various perspectives (e.g., time constraint, energy expenditure and biomechanics; Chen et al. 1994; Weerdesteyn et al. 2005). From a biomechanical point of view, step lengthening has been considered as stabilizing and, consequently, regarded as a safer strategy. The observation that the shorter-step adjustments (BW shifts) decreased steadily (larger error) with increasing task difficulty (smaller ARD), whereas for longer-step adjustments (FW shifts) the decrease was only significant for the condition that was most time critical (ARD 90 %), suggests that the longer-step strategy is also more robust against adverse influences associated with time pressure.

In contrast, for the older adults the longer-step responses (FW shifts) were only more accurate (smaller error) than the shorter-step responses (BW shifts) when time pressure was low (ARD 130 %; Fig. 3b). This supremacy of longer-step responses is consistent with the findings of Bank et al. (2011), who showed more adequate corrections for step adjustments following phase-delay than phase-advance shifts (comparable to FW and BW shifts, respectively, in our study) in older adults. Interestingly, however, this difference was not observed in older adults for the more time-critical conditions of the current study. This result may be associated with a ceiling effect for stepping error in the BW shift conditions. Whereas for FW shifts stepping error increased steadily with increasing time pressure demands, for BW shifts stepping error in the two least time-critical conditions was comparable, suggesting that in these conditions the maximum level of accuracy had been reached. This ceiling effect may be associated with the biomechanical aspects mentioned above and related to the observation by Chen et al. (1994) that tripping occurred only when older subjects tried excessive step shortening in an obstacle avoidance task. The steady increase in stepping error with increasing time pressure of FW shifts (involving longer-step adjustments) observed for the older participants was not found for the young participants. This result is in line with the findings of Chen et al. (1994), who showed that older adults experienced more difficulty compared to young adults to adopt the longer-step strategy when avoiding suddenly projected obstacles under higher time pressure.

Our finding that stepping error following SW shifts was larger in older adults than in young adults is consistent with Young and Hollands (2012), who showed that older adults make smaller step adjustments than young adults in response to lateral perturbations during visually cued walking. They attributed this deficit to age-related deterioration of visuomotor processing. However, decreased lateral stability with aging due to impaired neuromusculoskeletal function, like reduced hip abduction torque production and lateral trunk control (Mille et al. 2005), seems a likely contributing factor as well. In addition, we found that also for SW shifts the stepping error increased as a function of time pressure, consistent with the FW and BW shifts results (with largest errors for the shortest ARD) and that for both groups performance decreased when the cognitive task was performed concurrently.

One limitation of the study was that the single- and dual-task blocks were performed in a fixed order. We opted for this design feature to minimize the chance of demotivating effects (Potocanac et al. 2013, 2015) during the more difficult dual-task condition. Hence, we started the experiment with the easier single-task block followed by the more difficult dual-task block. This sequential design may have resulted in order effects, such as learning to perform the step adjustments. Hence, the effect of dual tasking on stepping error may be somewhat underestimated compared to a counterbalanced design. In addition, it is conceivable that the statistical results were affected by the limited sample size (15 persons per group). In particular, the absence of a significant difference between FW and BW shifts in older adults under higher time pressure conditions may be partly due to the limited sample size in combination with high variability of stepping error (especially for FW shifts). Finally, one may raise the question whether the step responses in the present study as obtained for visually cued treadmill walking are similar to those occurring in uncued walking. Visual attention to the area of landing could be different. In future experiments, it would therefore be useful to add an uncued walking condition in which only the to-be-shifted target appears from time to time. On the other hand, the current protocol has ecological validity since cued walking does occur naturally, for example, as when walking between patches of rain and deciding to adjust foot placement at the last instance (Moraes and Patla 2006).

In conclusion, we have demonstrated several age-related differences in step adjustments in response to sudden target shifts during visually cued walking. Compared to young adults, older adults made smaller step adjustment in response to FW and BW shifts under dual-task conditions, yielding larger stepping errors. In contrast to the posture-first principle, they appeared to sacrifice stepping performance in order to preserve their performance on the cognitive task, at least if one focuses on the stepping error for the shifted targets only. This prioritization of the cognitive task at the expense of the walking task may result in increased risk of falling in older adults in daily-life dual-tasking conditions (e.g., talking on a mobile phone when walking).

## Footnotes

1 To avoid confounding effects of speed on the manipulation of ARD and to be consistent with various previous studies (Hegeman et al. 2012; Potocanac et al. 2015), a fixed walking speed of 3 km/h was used.
